# Effects of Zishen Yutai pills combined with metformin on women with polycystic ovary syndrome undergoing in vitro fertilization

**DOI:** 10.1097/MD.0000000000039030

**Published:** 2024-08-02

**Authors:** Yu Zhang, Shan Cao, Jun-xia Liang, Shu-hong Hu, Xu-fang Guo, Shi Chun-jing, Li-na Ge

**Affiliations:** a Reproductive Department, Hebei Key Laboratory of Reproductive Medicine, Hebei Institute of Reproductive Health Science and Technology, Shijiazhuang, Hebei, China.

**Keywords:** in vitro fertilization and embryo transfer, metformin hydrochloride, polycystic ovary syndrome, Zishen Yutai pills

## Abstract

In this study, we analyzed the clinical efficacy of Zishen Yutai pills (ZSYTP) combined with metformin hydrochloride on infertile women diagnosed with polycystic ovary syndrome (PCOS) undergoing in vitro fertilization and embryo transfer (IVF-ET). Patients were assigned into 3 groups: the ZSYTP group (n = 50), the metformin group (n = 50), and the combination group (ZSYTP combined with metformin hydrochloride, n = 50), based on their respective and the indicated treatments before undergoing IVF-ET. Then, their glucose metabolism indices, sex hormone indices, traditional Chinese medicine (TCM) syndrome scores, and outcomes of IVF-ET were compared. Baseline characteristics were not significantly different between the 2 groups. After treatment, various parameters such as body mass index (BMI), fasting plasma glucose (FPG), fasting insulin (FIN), homeostatic model assessment of insulin resistance (HOMA-IR), luteinizing hormone (LH), estradiol (E2), follicle-stimulating hormone (FSH), testosterone (T) levels, and TCM syndrome scores were found to be reduced compared to pretreatment levels in both groups. Moreover, the improvement observed in the treatment group exceeded that of the control group. Specifically, the observation group displayed significantly lower gonadotropin (Gn) dosage and duration, as well as a reduced abortion rate compared to the control group. Furthermore, the observation group had higher numbers of obtained eggs, high-quality embryos, eggs obtained through IVF-ET, average transferred embryos, clinical pregnancy rate, and embryo implantation rate compared to the control group. Pretreatment with ZSYTP combined with metformin before IVF-ET in PCOS patients improves the outcome of IVF-ET.

## 1. Introduction

Polycystic ovary syndrome (PCOS), recognized as one of the primary causes of female infertility, represents a prevalent clinical reproductive endocrine and metabolic disorder among women of childbearing age.^[1]^ Clinically, PCOS is characterized by ovulatory dysfunction, hyperandrogenism, and insulin resistance.^[2]^ Despite symptomatic treatment, spontaneous remission or resolution with medication is not typically achieved in PCOS patients. Despite the current low probability of pregnancy associated with this condition, individuals of childbearing age affected by PCOS often express a strong desire to conceive.^[3]^ It is noteworthy that with the progression of assisted reproductive technology, in vitro fertilization and embryo transfer (IVF-ET) have emerged as an effective treatment for PCOS related infertility.^[3]^ Nonetheless, achieving optimal fertilization rates, embryo implantation rates, and ultimately successful pregnancy outcomes remains challenging with IVF-ET. Therefore, patients may still experience pregnancy failure, significantly impacting their quality of life as well as their physical and mental well-being. Hence, identifying appropriate and efficacious therapeutic interventions is imperative to enhance ovarian function and alleviate clinical symptoms in PCOS patients undergoing IVF-ET.

Zishen Yutai pills (ZSYTP) are Chinese patent medicines formulated comprising various medicinal herbs, including dodder seeds, villous amomum fruits, prepared Rehmannia root, ginseng, Chinese Taxillus herb, ass hide glue, Fleece flower root, argy wormwood leaf, Morinda root, Largehead atractylodes rhizome, Tangshen, Degelatined deer-horn, Barbary wolfberry fruit, Himalayan teasel root, and Eucommia bark.^[4]^ In terms of pharmacological properties, ZSYTP has shown benefits in pregnancy, prevention and treatment of threatened and habitual abortions, improvement of ovarian reserve, and regulation of menstrual cycles.^[5]^ Additionally, due to their ability to stimulate hormone secretion and ovulation in the ovaries, ZSYTP contributes to increased ovulation rates, enhanced pregnancy rates, and reduced abortion rates. Recently, ZSYTP has been utilized to enhance pregnancy outcomes in PCOS patients undergoing IVF-ET. However, Chinese medicine treatments alone may have limited efficacy in significantly improving pregnancy outcomes, indicating the need for further improvements in enhancing the pregnancy rate in patients.^[6]^

Metformin hydrochloride is widely used as an insulin sensitizer for the treatment of PCOS due to its ability to alleviate hyperinsulinemia and hyperandrogenism associated with the condition, thereby facilitating ovulation.^[7]^ Metformin also acts synergistically to reduce levels of luteinizing hormone (LH) and androgens in the bloodstream.^[8]^ However, the oral administration of metformin alone in PCOS treatment typically provides only temporary relief from clinical symptoms. Notably, there are no prior reports on the combined application of ZSYTP and metformin hydrochloride in infertile women with PCOS undergoing IVF-ET for assisted reproduction. Therefore, this study aimed to assess the clinical efficacy of ZSYTP combined with metformin hydrochloride in infertile PCOS patients undergoing IVF-ET, adopting an integrated Chinese-Western therapeutic approach.

Herein, we hypothesized that the combined administration of ZSYTP and metformin would improve pregnancy outcomes in infertile PCOS patients undergoing IVF-ET. The primary study outcomes included ovulation induction status and IVF-ET outcomes, while the secondary outcomes encompassed body mass index (BMI), glucose metabolism indices, sex hormone levels, and traditional Chinese medicine (TCM) syndrome scores. Overall, this study aims to provide a foundational understanding for the clinical application of this combined therapeutic approach.

## 2. Materials and methods

### 2.1. General information

**Figure 1.**
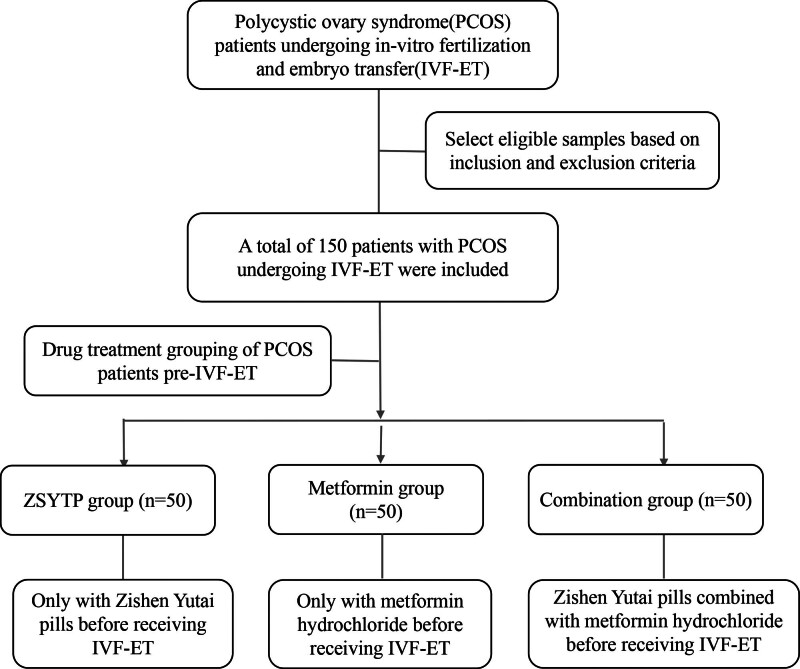
Flow chart of sample screening. IVF-ET = in vitro fertilization and embryo transfer, PCOS = polycystic ovary syndrome, ZSYTP = Zishen Yutai pills.

Based on the study inclusion and exclusion criteria, we retrospectively retrieved the data of PCOS patients who underwent IVF-ET at the Hebei Institute of Reproductive Health Science and Technology between April 2019 and April 2022 from the hospital records. They were categorized into 3 groups: the ZSYTP group (n = 50; mean age = 30.2 years), the metformin group (n = 50; mean age = 30.0 years), and the combination group (n = 50; mean age = 31.5 years), based on their respective treatments prior to IVF-ET at the treating physician’s discretion. The study flow chart is shown in Figure 1. The study protocol was approved by the Ethics Committee of the Hebei Institute of Reproductive Health Science and Technology.

The inclusion criteria were: patients diagnosed with PCOS according to the Rotterdam 2003 criteria recommended by the European Society of Human Reproduction and Embryology (ESHRE)/American Society of Reproductive Medicine (ASRM),^[9]^ meeting at least 2 of the following 3 criteria: (Oligoovulation and/or anovulation; secondly clinical manifestations and/or biochemical indicators of hyperandrogenism; and finally polycystic ovarian changes via B ultrasound demonstrating at least 1 ovarian antral follicle (diameter 2–9 mm) count ≥ 12, and/or ovarian volume > 10 mL); patients presenting with infertility and a negative result on a urine pregnancy test prior to treatment; patients aged between 22 and 40 years, with a desire for fertility, and a BMI ranging from 24 to 30 kg/m^2^; patients lacking organic reproductive system diseases and demonstrating bilateral tubal patency. The exclusion criteria were: presence of congenital gonadal dysgenesis, reproductive organ malformation, uterine amenorrhea, or history of uterine and ovarian surgery; occurrence of congenital adrenal hyperplasia, Cushing syndrome, thyroid lesions, or tumors; diagnosis of hyperprolactinemia or other endocrine disorders; and insufficient or absent clinical information.

All included patients were diagnosed with a deficiency syndrome characterized by symptoms including menstrual irregularities (gradual amenorrhea or menstrual cycle disorder), light-colored menstrual blood with low viscosity, dizziness, tinnitus, lower back and knee pain and weakness, sensitivity to cold, and cold extremities. Secondary symptoms included decreased libido and an increased volume of vaginal discharge with low viscosity. The physical examination showed a pale tongue with a white coating and a deep pulse. Patients were diagnosed if they exhibited a combination of these primary symptoms and physical examination findings according to the *Guiding Principles of Clinical Research on New Herbs (Trial*).^[10]^

### 2.2. Treatment methods

#### 2.2.1. Pretreatment strategies

In the combination group, patients underwent pretreatment with a combination of ZSYTP and metformin hydrochloride prior to IVF-ET. ZSYTP (manufactured by Guangzhou Baiyunshan Zhongyi Pharmaceutical Co., Ltd.; approved by the State Food and Drug Administration (SFDA) with approval number Z44020008; strength: 60 g/bottle) were orally administered for 3 consecutive courses, with each course lasting 1 month. The dosage regimen involved administration 3 times daily, with 5 g/dose.^[10]^ Importantly, administration was suspended during menstruation to avoid any possible adverse effects that might arise from hormonal fluctuations during this period. Metformin hydrochloride (manufactured by Jinan Hengji Pharmaceutical Co., Ltd.; SFDA approval number: H37021405; strength: 0.25 g/tablet) was orally administered for 3 consecutive courses, with each course lasting 1 month. The prescribed regimen involved administration 3 times daily, with a dosage of 5 g/dose, with administration suspended during menstruation.^[6]^

In the ZSYTP group, patients underwent pretreatment with ZSYTP alone before IVF-ET, following the same administration protocol as in the combination group, which entailed continuous administration over 3 consecutive courses, with each course lasting 1 month, with the administration suspended during menstruation.

Similarly, in the metformin group, patients received pretreatment with metformin hydrochloride alone before IVF-ET, adhering to the same administration protocol as outlined for the combination group: 3 consecutive courses, each lasting 1 month.

#### 2.2.2. In vitro fertilization and embryo transfer

Ovulation induction in the enrolled patients followed a conventional protocol. IVF-ET was performed in the cycle immediately following the completion of the 3-month treatment regimen. Briefly, pituitary downregulation was achieved by administering long-acting gonadotropin-releasing hormone agonists intramuscularly on day 21 of the menstrual cycle. Upon reaching the desired level of suppression, gonadotropin (Gn) was administered to stimulate ovarian activity. The Gn dosage was adjusted on the 5th day of continuous administration based on transvaginal ultrasound findings and hormone level fluctuations. Ovulation was triggered when at least 3 follicles measured ≥17 mm or 2 follicles measured ≥18 mm in diameter, with an injection of 5000 IU of recombinant human chorionic gonadotropin administered that evening. Oocyte retrieval was conducted 36 hours later, assisted by transvaginal ultrasonography, followed by embryo transfer under transabdominal ultrasound guidance, 3 days post-retrieval. Subsequently, the patients received standard luteal phase support. A serum β-human chorionic gonadotropin test was conducted 12 to 14 days after embryo transfer, and a transvaginal ultrasound examination was performed 4 weeks post-transfer to assess for clinical pregnancy, indicated by the presence of a gestational sac.

### 2.3. Outcome measures

1. BMI and glucose metabolism indices: BMI assessments were conducted before and after treatment in the 3 cohorts. Fasting Plasma Glucose (FPG) and Fasting Insulin (FIN) levels were measured, and Homeostatic Model Assessment of Insulin Resistance (HOMA-IR) was computed using the formula: HOMA-IR = (FIN × FPG)/22.5, based on glucose tolerance and insulin release tests.
2. Sex hormone indices: Serum levels of LH, follicle-stimulating hormone (FSH), estradiol (E2), and testosterone (T) were quantified using enzyme-linked immunosorbent assay on the 2nd to 5th day of menstruation posttreatment.
3. TCM syndrome scores: TCM syndrome scores were assessed both pre- and posttreatment in each study group, utilizing a designated scale tailored to evaluate symptom amelioration within the 2 groups. The scale included 9 items: menstrual cycle regularity, menstrual volume, menstrual blood appearance, dizziness and tinnitus, soreness and weakness of the lower back and knees, sensitivity to cold, cold extremities, reduced sexual desire, and vaginal discharge. Scores ranged from 0 to 3, indicating the severity of each symptom: 0 for none, 1 for mild, 2 for moderate, and 3 for severe; higher scores indicated more severe symptoms. For criteria with multiple elements, such as soreness and weakness of the lower back and knees, each element was assessed individually, and an average score was calculated to represent the overall severity of the criterion.
4. Ovulation induction: The duration and dosage of Gn use, number of oocytes retrieved, and number of high-quality embryos were recorded.
5. IVF-ET outcomes: The number of oocytes retrieved, average number of embryos transferred, clinical pregnancy rate, embryo implantation rate, and abortion rate were collected from the 3 groups of patients.

### 2.4. Statistical analysis

Statistical analysis was conducted using SPSS 22.0. The normality of measurement data was assessed via the Shapiro–Wilk test. Measurement data conforming to a normal distribution are expressed as mean ± standard deviation (SD). Paired *t* tests were utilized to compare groups before and after treatment, while 1-way analysis of variance was used to compare 3 groups. Enumeration data are presented as n (%), with the chi-squared test or Fisher exact test employed for comparisons. A significance level of *P* < .05 was considered statistically significant.

## 3. Results

### 3.1. Clinical baseline information of the patients

In this study, 150 PCOS patients who underwent IVF-ET were found eligible, and their clinical data were collected and retrospectively assessed. Data analysis showed no statistically significant differences among the 3 groups in terms of age, duration of PCOS, presence of primary infertility, and total follicle count (*P* > .05, Table 1).

**Table 1:** Clinical baseline information of the patients.

Groups	ZSYTP group (n = 50)	Metformin group (n = 50)	Combination group (n = 50)	T/χ^2^	*P*
Age (yr)*	30.18 ± 5.84	29.96 ± 5.97	31.54 ± 4.90	1.172	.313
Duration of PCOS (yr)*	3.35 ± 0.44	3.27 ± 0.50	3.18 ± 0.59	1.344	.264
Presence of primary infertility (%)†				0.450	.798
No	13(26.0)	15(30.0)	16(32.0)		
Yes	37(74.0)	35(70.0)	34(68.0)		
Total follicle count*	19.96 ± 3.27	19.68 ± 3.33	19.70 ± 2.94	0.120	.887

ANOVA = analysis of variance, PCOS = polycystic ovary syndrome, ZSYTP = Zishen Yutai pills.

* Indicates that the parameter was measurement data (mean ± standard deviation) that conform to normal distribution and the ANOVA was used for comparison between the 3 groups.

† Indicates that the parameter was enumeration data (n, %) and χ^2^ test was used for comparison.

### 3.2. Comparison of BMI and glucose metabolism indexes before and after treatment

As shown in Table 2, we found no significant differences in BMI, FPG, FIN, and HOMA-IR between the 3 groups before treatment (*P* > .05). However, compared with those before treatment, BMI, FPG, FIN, and HOMA-IR in the 3 groups were significantly lower after treatment (*P* < .05). In addition, BMI, FPG, FIN, and HOMA-IR in the combination group were significantly lower than those in the ZSYTP and metformin groups after treatment (*P* < .05).

**Table 2:** Comparison of body mass index and glucose metabolism indexes before and after treatment.

Groups		ZSYTP group (n = 50)	Metformin group (n = 50)	Combination group (n = 50)
BMI (kg/m^2^)	Before treatment	26.64 ± 1.22	26.78 ± 0.88	26.32 ± 1.37
	After treatment	24.95 ± 1.09***	25.27 ± 1.19***	22.15 ± 1.20*^,^**^,^***
FPG (mmol/L)	Before treatment	5.31 ± 0.20	5.32 ± 0.23	5.23 ± 0.51
	After treatment	4.97 ± 0.17***	5.03 ± 0.20***	4.52 ± 0.29*^,^**^,^***
FIN (μU/mL)	Before treatment	13.58 ± 0.81	13.76 ± 1.11	13.86 ± 1.39
	After treatment	11.48 ± 0.76***	11.68 ± 1.17***	9.47 ± 1.24*^,^**^,^***
HOMA-IR	Before treatment	3.20 ± 0.17	3.25 ± 0.31	3.20 ± 0.17
	After treatment	2.53 ± 0.14***	2.61 ± 0.27***	1.89 ± 0.20*^,^**^,^***

The parameter was measurement data (mean ± standard deviation).

BMI = body mass index, FIN = fasting insulin, FPG = fasting plasma glucose, HOMA-IR = homeostatic model assessment of insulin resistance, ZSYTP = Zishen Yutai pills.

* *P* < .05 versus ZSYTP group.

** *P* < .05 versus metformin group.

*** *P* < .05 versus before treatment.

### 3.3. Comparison of sex hormone levels after treatment between the 3 groups

As shown in Table 3, there was no significant difference in LH, FSH, E2, and T levels among the 3 groups before treatment (*P* > .05). However, the levels of LH, E2 and T in the 3 groups were significantly decreased after treatment compared with before treatment (*P* < .05). Notably, FSH levels were not significantly different before and after treatment (*P* > .05). The differences in LH, E2 and T levels between the 3 groups after treatment were statistically significant (*P* < .05; Table 3).

**Table 3:** Comparison of sex hormone levels before and after treatment between the 3 groups.

Groups		ZSYTP group (n = 50)	Metformin group (n = 50)	Combination group (n = 50)
	Before treatment	16.29 ± 2.03	16.18 ± 1.93	15.98 ± 1.98
LH (IU/L)	After treatment	12.28 ± 1.31***	12.67 ± 1.08***	9.63 ± 1.01*^,^**^,^***
FSH (U/L)	Before treatment	5.53 ± 0.26	5.54 ± 0.22	5.51 ± 0.24
	After treatment	5.46 ± 0.26	5.48 ± 0.29	5.45 ± 0.28
E2 (pmol/L)	Before treatment	122.89 ± 5.49	123.04 ± 5.76	122.67 ± 7.35
	After treatment	117.46 ± 4.96***	118.92 ± 4.60***	106.31 ± 5.36*^,^**^,^***
T (nmol/L)	Before treatment	17.43 ± 2.88	17.81 ± 2.73	17.60 ± 3.00
	After treatment	14.57 ± 2.46***	15.21 ± 2.58***	12.54 ± 2.40*^,^**^,^***

The measurement data was expressed as mean ± standard deviation.

E2 = estradiol, FSH = follicle-stimulating hormone, LH = luteinizing hormone, T = testosterone, ZSYTP = Zishen Yutai pills.

* *P* < .05 versus ZSYTP group.

** *P* < .05 versus metformin group.

*** *P* < .05 versus before treatment.

### 3.4. Comparison of TCM syndrome scores before and after treatment between the 3 groups

Before treatment, there was no significant difference between the 2 groups in the scores for various TCM symptoms (*P* > .05). Compared with those before treatment, all TCM symptom scores of the 3 groups significantly decreased after treatment (*P* < .05), and this reduction was more significant in the combination group than in the ZSYTP and metformin groups (*P* < .05; Table S1, Supplemental Digital Content, http://links.lww.com/MD/N397).

### 3.5. Comparison of ovulation induction between the 3 groups

Compared with the ZSYTP or metformin group, the combination group had a significantly shorter duration of Gn use (9.78 ± 3.42 days vs 13.32 ± 3.05 days, 13.42 ± 3.53 days), lower dosage of Gn use (21.80 ± 5.07 vials vs 29.96 ± 9.28 vials, 30.92 ± 8.57 vials), larger number of oocyte retrieval (11.52 ± 2.88 vs 9.56 ± 2.73, 9.42 ± 3.28) and more high-quality embryos (3.86 ± 0.93 vs 2.94 ± 1.19, 2.90 ± 0.97; *P* < .05; Table S2, Supplemental Digital Content, http://links.lww.com/MD/N398).

### 3.6. Comparison of outcomes of in vitro fertilization and embryo transfer between the 3 groups

The combination group showed a significantly higher number of oocytes retrieved (46 vs 32, 32), average number of embryos transferred (1.96 ± 0.45 vs 1.18 ± 0.39, 1.28 ± 0.45), clinical pregnancy rate (70.0% vs 46.0%, 44.0%), and embryo implantation rate (66.0% vs 42.0%, 38.0%), but a much lower abortion rate than the other 2 groups (6.0% vs 24.0%, 24.0%; *P* < .05; Table S3, Supplemental Digital Content, http://links.lww.com/MD/N399).

## 4. Discussion

This present study found no significant differences in baseline characteristics among the 3 groups. Following medication before IVF-ET, we found that BMI, FPG, FIN, HOMA-IR, LH, E2, FSH, and T levels decreased in all groups compared to pretreatment levels, with a significantly greater reduction observed in the combination group compared to the ZSYTP or metformin group. Moreover, TCM symptom scores decreased in both groups posttreatment, with a more pronounced improvement observed in the combination group compared to the ZSYTP or metformin group. Regarding ovulation and IVF-ET outcomes, the combination group exhibited significantly lower duration and dosage of Gn use and miscarriage rate compared to the other groups. Additionally, the combination group demonstrated significantly higher numbers of retrieved eggs, high-quality embryos, eggs obtained in IVF-ET, embryos transferred, clinical pregnancy rate, and embryo implantation rate compared to the ZSYTP or metformin group. Overall, our findings suggest that pretreatment with ZSYTP combined with metformin hydrochloride before IVF-ET in patients with PCOS can substantially improve ovulation and IVF-ET outcomes, demonstrating significant clinical efficacy.

PCOS is a genetically related endocrine disorder characterized by various clinical manifestations and significantly impacting women’s physical and psychological well-being worldwide.^[11]^ In cases where the initial interventions to treat PCOS fail to achieve pregnancy, IVF-ET becomes necessary. China is a global leader in IVF-ET treatments, with over 300,000 cycles conducted annually.^[12]^ Concerns have been raised about the use of superovulation medications during IVF-ET, which may improve ovum maturation but also disrupt normal female physiological processes. This disruption can potentially lead to hormonal imbalances and further result in metabolic and renal dysfunction.^[13,14]^ Older patients undergoing IVF-ET often have prolonged infertility histories and are particularly susceptible to these imbalances, which can compromise the stability of pregnancy.^[15]^ In addition, emotional stress can exacerbate these conditions, further increasing the risk of miscarriage.^[16]^ In assisting IVF-ET, treatment should specifically target patients’ symptoms and provide tailored therapeutic interventions. Additionally, TCM aims to harmonize bodily functions by enhancing overall physiological balance, improving energy levels, supporting organ function, and promoting blood circulation.^[17,18]^

The Zishen Yutai pill, formulated by Professor Luo Yuankai from Guangdong, China, is renowned for its efficacy in gynecology.^[19]^ This prescription is distinguished by its diverse therapeutic effects, including supporting kidney function, enhancing metabolic processes, boosting overall energy, and promoting blood health. Currently, Zishen Yutai pills are widely used in the treatment of gynecological disorders, particularly in managing conditions that affect fertility and pregnancy.^[20]^ Furthermore, formulated as Chinese patent medicines for oral administration, ZSYTP are favored for their convenience and minimal adverse reactions, earning widespread application in clinical practice.^[21]^

Metformin hydrochloride, a well-established hypoglycemic agent used in PCOS treatment for over 5 decades, reduces hepatic glycogen production, enhances peripheral tissue insulin sensitivity, and lowers compensatory hyperinsulinemia, ultimately decreasing androgen production.^[22]^ It also suppresses adipocytokines and serum inflammatory factors, mitigating insulin resistance,^[23]^ and inhibits androgen synthase cytochrome P450c17a (CYP17) expression in theca cells, reducing androstenedione secretion and inhibiting progesterone and E2 secretion by granulosa cells.^[24]^ Clinical studies have demonstrated its effectiveness in reducing LH, FIN, and T levels, promoting ovulation, and improving conception rates in PCOS patients by alleviating local insulin resistance in the endometrium and regulating serum inflammatory factor levels, such as C-reactive protein and tumor necrosis factor-α.^[25]^

In this study, an analysis was conducted on 150 patients with PCOS who underwent IVF-ET. Briefly, posttreatment BMI, glucose metabolism indices (FPG, FIN, and HOMA-IR), and sex hormone levels (LH, E2, and T) in the 3 groups were significantly lower than pretreatment levels, and all TCM syndrome scores notably improved. Importantly, the treatment effect in the combination group (patients receiving ZSYTP combined with metformin hydrochloride) was markedly superior to that in the ZSYTP or metformin group. Overall, ZSYTP combined with metformin hydrochloride significantly reduced BMI, increased insulin sensitivity, and improved TCM symptoms in infertile PCOS patients, consistent with findings reported by Naderpoor et al^[26]^ and Oner and Muderris.^[27]^ It is well recognized that obesity is closely associated with the development of PCOS. Previous studies have suggested that metformin hydrochloride may decrease fat synthesis, reduce body weight, improve BMI, and enhance the waist-to-arm ratio by inhibiting acetyl-CoA hydroxylase function through the AMP-activated protein kinase (AMPK) pathway.^[28,29]^ Additionally, ZSYTP can regulate FSH, LH, and E2 levels, thus exerting comprehensive regulatory effects, improving the internal environment and ovarian secretion, and alleviating TCM symptoms in PCOS patients.^[30]^ Research has indicated that in PCOS patients, the decrease in insulin levels induced by metformin alleviates insulin’s stimulatory effect on LH, leading to reduced LH levels and LH/FSH ratio, improvement of hormonal imbalances, and promotion of follicular development and ovulation.^[31]^ These findings are consistent with the results of our study. Additionally, metformin has been reported to significantly reduce the cancelation rate of transplantation and E2 levels, decrease the duration and dosage of Gn stimulation, and notably increase the clinical pregnancy rate.^[32,33]^ In this study, both the duration and dosage of Gn use and the abortion rate in the combination group were significantly lower than those in the ZSYTP and metformin groups. Moreover, the combination group also demonstrated substantially higher numbers of retrieved oocytes, high-quality embryos, oocytes retrieved by IVF-ET, average number of embryos transferred, clinical pregnancy rate, and embryo implantation rate compared to the group receiving medication alone. These findings suggest that the combination of ZSYTP with metformin hydrochloride can significantly enhance the pregnancy outcomes of PCOS patients undergoing IVF-ET. The mechanism of action may be attributed to the pharmacological activity of each ingredient in ZSYTP and the relevant targets of metformin hydrochloride.

This study had some limitations. Firstly, the sample size of 150 patients, though adequate for preliminary findings, is relatively small. Larger, multicenter studies are necessary to confirm the generalizability of our results. Secondly, our study population was limited to patients from a single institute, potentially introducing selection bias. Diverse populations from different geographic and clinical settings should be included in future studies to validate our findings. Third, the follow-up period in our study was limited to the immediate IVF-ET outcomes. Long-term follow-up studies are needed to assess the sustainability of the treatment benefits and potential long-term effects.

## 5. Conclusion

Pretreatment with ZSYTP combined with metformin hydrochloride in women with PCOS prior to IVF-ET demonstrates significant improvements in ovarian function, abnormal glucose metabolism, and TCM-related symptoms. Additionally, it reduces patient BMI, the duration and dosage of Gn use, and enhances IVF-ET outcomes. However, these findings should be interpreted with caution. Further validation in larger, multicenter, randomized controlled trials is necessary to establish the clinical efficacy and safety of this combined therapeutic approach.

## Author contributions

**Conceptualization:** Yu Zhang, Shan Cao.

**Data curation:** Jun-xia Liang, Shu-hong Hu, Xu-fang Guo, Chun-jing Shi, Li-na Ge.

**Formal analysis:** Jun-xia Liang, Shu-hong Hu, Xu-fang Guo, Chun-jing Shi, Li-na Ge.

**Writing – original draft:** Yu Zhang, Shan Cao.

**Writing – review & editing:** Yu Zhang, Shan Cao, Jun-xia Liang, Shu-hong Hu, Xu-fang Guo, Chun-jing Shi, Li-na Ge.

## Supplementary Material






