# Supplementary material for: Effects of Zishen Yutai pills combined with metformin on women with polycystic ovary syndrome undergoing in vitro fertilization
Source: Medicine (Baltimore). 2024 Aug 2;103(31):e39030. doi: 10.1097/MD.0000000000039030 (PMC11296412; doi:10.1097/MD.0000000000039030)
Supplement: Supplementary file 1 [file medi-103-e39030-s001.docx]

**Table S1** Comparison of traditional Chinese medicine symptom scores before and after treatment between the three groups

|  | ZSYTP group  (n = 50) | Metformin group  (n = 50) | Combination group  (n = 50) | F | *P* |
| --- | --- | --- | --- | --- | --- |
| Before treatment | 19.02±2.33 | 18.86±1.65 | 18.68±2.29 | 0.323 | 0.724 |
| After treatment | 5.74±1.51 | 5.62±1.34 | 5.18±1.73^*#^ | 1.843 | 0.162 |
| T | 32.619 | 40.401 | 32.586 |  |  |
| *P* | < 0.001 | < 0.001 | < 0.001 |  |  |

The measurement data was expressed as mean ± standard deviation. Zishen Yutai pills (ZSYTP) ^*^*P* < 0.05 *vs*. ZSYTP group; ^#^*P* < 0.05 *vs*. Metformin group
