# Supplementary material for: Effects of Zishen Yutai pills combined with metformin on women with polycystic ovary syndrome undergoing in vitro fertilization
Source: Medicine (Baltimore). 2024 Aug 2;103(31):e39030. doi: 10.1097/MD.0000000000039030 (PMC11296412; doi:10.1097/MD.0000000000039030)
Supplement: Supplementary file 2 [file medi-103-e39030-s002.docx]

**Table S2** Comparison of ovulation induction between the three groups

| Groups | ZSYTP group (n = 50) | Metformin group  (n = 50) | Combination group  (n = 50) | F | *P* |
| --- | --- | --- | --- | --- | --- |
| Duration of Gn use (day) | 13.32±3.05 | 13.42±3.53 | 9.78±3.42^*#^ | 19.222 | < 0.001 |
| Dosage of Gn use (vial) | 29.96±9.28 | 30.92±8.57 | 21.80±5.07^*#^ | 20.343 | < 0.001 |
| Number of eggs obtained | 9.56±2.73 | 9.42±3.28 | 11.52±2.88^*#^ | 7.800 | 0.001 |
| Number of high-quality embryos | 2.94±1.19 | 2.90±0.97 | 3.86±0.93^*#^ | 13.778 | < 0.001 |

The measurement data was expressed as mean ± standard deviation. Zishen Yutai pills (ZSYTP) ^*^*P* < 0.05 *vs*. ZSYTP group; ^#^*P* < 0.05 *vs*. Metformin group.
