# Supplementary material for: Effects of Zishen Yutai pills combined with metformin on women with polycystic ovary syndrome undergoing in vitro fertilization
Source: Medicine (Baltimore). 2024 Aug 2;103(31):e39030. doi: 10.1097/MD.0000000000039030 (PMC11296412; doi:10.1097/MD.0000000000039030)
Supplement: Supplementary file 3 [file medi-103-e39030-s003.docx]

**Table S3** Comparison of the outcomes of in-vitro fertilization and embryo transfer between the three groups

| Groups | ZSYTP  Group (n = 50) | Metformin  group (n = 50) | Combination  group (n = 50) | F/χ^2^ | *P* |
| --- | --- | --- | --- | --- | --- |
| Number of eggs obtained | 32 | 32 | 46^*#^ | 220.281 | < 0.001 |
| Average number of embryos transferred | 1.18±0.39 | 1.28±0.45 | 1.96±0.45^*#^ | 48.356 | < 0.001 |
| Clinical pregnancy rate | 23(46.0) | 22(44.0) | 35(70.0)^*#^ | 8.411 | 0.015 |
| Embryo implantation rate (%) | 21(42.0) | 19(38.0) | 33(66.0)^*#^ | 9.180 | 0.010 |
| Abortion rate (%) | 12(24.0) | 12(24.0) | 3(6.0)^*#^ | 7.317 | 0.026 |

Zishen Yutai pills (ZSYTP). ^*^*P* < 0.05 *vs*. ZSYTP group; ^#^*P* < 0.05 *vs*. Metformin group.
